# Influence of an Interdisciplinary Re-employment Programme Among Unemployed Persons with Mental Health Problems on Health, Social Participation and Paid Employment

**DOI:** 10.1007/s10926-017-9704-3

**Published:** 2017-04-10

**Authors:** Bouwine E. Carlier, Merel Schuring, Alex Burdorf

**Affiliations:** Department of Public Health, Erasmus University Medical Center, P.O. Box 2040, 3000 CA Rotterdam, The Netherlands

**Keywords:** Unemployment, Mental health, Employment, Re-employment programme, Propensity score

## Abstract

*Purpose* To evaluate the influence of an interdisciplinary re-employment programme on labour force participation and perceived health among unemployed persons with common mental health problems. In addition, the influence of entering paid employment on self-rated physical health and mental health was investigated. *Methods* In this quasi-experimental study with 2 years follow up, 869 persons were enrolled after referral to an interdisciplinary re-employment programme (n = 380) or regular re-employment programme (n = 489). The propensity score technique was used to account for observed differences between the intervention and control group. The intervention programme was provided by an interdisciplinary team, consisting of mental health care professionals as well as employment specialists. Mental health problems were addressed through cognitive counselling and individual tailored job-search support was provided by an employment professional. Primary outcome measures were paid employment and voluntary work. Secondary outcome measures were self-rated mental and physical health, measured by the Short Form 12 Health Survey, and anxiety and depressive symptoms, measured by the Kessler Psychological Distress Scale. Changes in labour force participation and health were examined with repeated-measures logistic regression analyses by the generalized estimating equations method. *Results* The interdisciplinary re-employment programme did not have a positive influence on entering employment or physical or mental health among unemployed persons with mental health problems. After 2 years, 10% of the participants of the intervention programme worked fulltime, compared to 4% of the participants of the usual programmes (adjusted OR 1.65). The observed differences in labour force participation were not statistically significant. However, among persons who entered paid employment, physical health improved (+16%) and anxiety and depressive symptoms decreased (−15%), whereas health remained unchanged among persons who continued to be unemployed. *Conclusions* Policies to improve population health should take into account that promoting paid employment may be an effective intervention to improve health. It is recommended to invest in interdisciplinary re-employment programmes with a first place and train approach.

**Electronic supplementary material:**

The online version of this article (doi:10.1007/s10926-017-9704-3) contains supplementary material, which is available to authorized users.

## Introduction

In the past two decades employment has received growing attention as an important determinant of health inequalities [1]. Unemployed persons have a higher prevalence of illness, disability [2, 3] and psychological disorders [4, 5]. In addition, unemployed persons have a lower healthy life expectancy (reduced length of time individuals spend in good health) and a higher mortality compared to employed persons [6, 7]. Two mechanisms may contribute to these inequalities in health. The selection mechanism may act through two different pathways: unemployed persons with a poor health are less likely to enter the workforce and employed persons with poor health are more likely to become unemployed [8–11]. The causation mechanism may also act in two different ways. Becoming unemployed has a negative effect on health. It is consistently demonstrated that individuals becoming unemployed develop poorer mental health [12–14] as well as poor self-rated health [3]. On the other hand, re-employment may be beneficial for health, particularly for mental health [5]. Several observational studies [15–18] and a recent review [19] showed a positive effect of re-employment on health, most notably on depression and mental health.

Consequently, interventions promoting employment may protect against the adverse health effects of unemployment and may improve health among unemployed persons, especially among persons with (mental) health problems. However, unemployed persons with a poor (mental) health have more difficulty finding paid employment. Therefore, effective re-employment programs are needed to improve labour force participation and health among unemployed persons with mental health problems. The individual placement and support (IPS) approach for persons with severe mental health problems consists of a combination of treatment, rapid job placement, and job-coaching in the new job [20]. This integrated approach of health and employment services resulted in higher rates of competitive employment, fewer days to the first competitive job and more hours and weeks worked among persons with severe mental disorders [21–23] As crucial factor for success is considered the alignment of the client’s preferences and work possibilities with individualized job support at workplaces with receptive employers [24].

There is also some evidence to support the use of cognitive counselling on personal development and preparedness against setbacks during the job-search process [15]. Job search interventions including motivation enhancement via cognitive counselling showed to be effective in finding paid employment [25]. A recent study showed that unemployed persons with a positive attitude towards job-search and a high self-efficacy in searching for a job were more likely to search actively and also to actually find paid employment [26].

With this evidence in mind, a re-employment programme was developed in the city of Rotterdam, the Netherlands, for long-term unemployed persons with mental health problems. Employment professionals and mental health professionals worked together in an interdisciplinary team. Mental health problems that were a barrier to enter paid employment, were addressed by a psychologist through cognitive counselling. Simultaneously, employment professionals provided individual tailored job-search support taking into account possibilities and limitations of the client. The purpose of the programme was to increase labour force participation and improve mental health among unemployed persons with health problems. The first aim of the current study was to evaluate the influence of this re-employment programme on entering employment as well as physical and mental health of unemployed persons with common mental health problems. The second aim was to evaluate the influence of entering paid employment on physical health and mental health.

## Methods

### Study Design and Population

The study was designed as a quasi-experiment by comparing an interdisciplinary re-employment programme with regular re-employment programmes. Randomization was not feasible since the interdisciplinary re-employment programme was already implemented within the organization. The propensity score matching technique was used as alternative research design to evaluate effectiveness of interventions when a randomized controlled trial is not feasible [27, 28]. The formal assumption is that all differences in treatment and control group are due to observable characteristics. The propensity score is defined as the conditional probability of treatment, given these characteristics [29, 30]. Eligibility criteria for participants in the study were: (1) receiving social security benefits due to unemployment, (2) capable of employment according to the social service officer and (3) recently referred to a re-employment programme by the local Employment Center.

### Allocation to the Treatment and Control Group

**Fig. 1:**
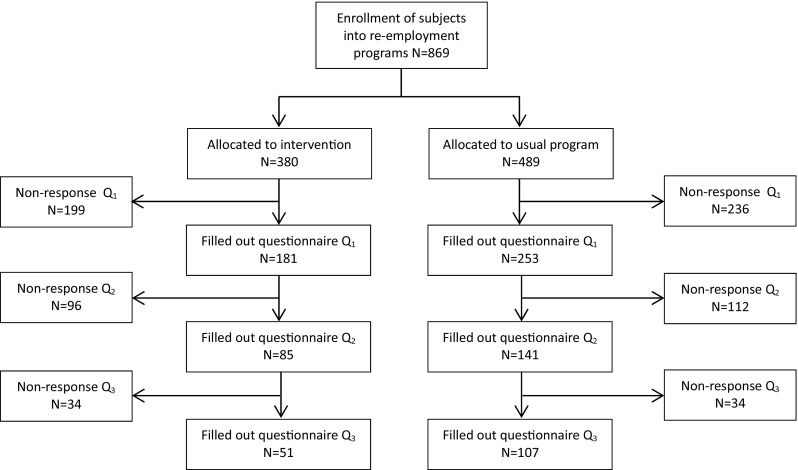
Flow chart filled out questionnaires among those how received the intervention or received usual care programs

From March 2011 until August 2014, persons who were recently referred to an interdisciplinary re-employment programme or a regular re-employment programme, were approached by the researcher for participation in the study. Professionals of the local Employment Center referred persons to an interdisciplinary re-employment programme (treatment group) or a regular re-employment programme (control group). When employment professionals suspected mental health problems, a psychologist was involved to confirm the presence of mental health problems. Persons with common mental health problems, such as anxiety or depression, were preferably referred to the interdisciplinary re-employment programme. However, professionals of the Employment Center were often not aware of the presence of mental health problems. Therefore, among those who were referred to regular re-employment programmes, (undiagnosed) mental health problems may also be present. Figure 1 shows the diagram of the flow of participants through the phases of the study. In total, 380 persons were allocated to the intervention programme and 489 persons to usual programmes.

### Intervention

Professionals from the mental health services and the employment services worked together in an interdisciplinary team to guide persons to paid employment. The programme started with an assessment by the interdisciplinary team including employment specialists, social workers and mental health professionals. Barriers for entering paid employment, such as psychological problems or debts, were addressed. Psychological resources for entering paid employment, such as self-confidence and self-efficacy, were enhanced by coaching and cognitive counselling. The cognitive counselling focussed on general- as well as job-search self-efficacy beliefs. Employment activities, such as job-search support and temporary voluntary work placement, were tailored to meet the specific needs of each unemployed person. The maximum duration of the programme was 2 years.

### Usual Re-employment Programmes

There was a large variety of different employment programmes, including voluntary work programmes, physical activity programmes and life coaching. However, in none of these programmes persons were guided towards paid employment by an interdisciplinary team including mental health care professionals. The voluntary work programme facilitated placement on temporary voluntary jobs. The physical activity programme consisted of 2-weekly physical activity (in groups or individual) in combination with vocational training aimed at re-employment. The life coaching programme was carried out by a case manager which supported persons in achieving personal goals on different domains of life, for example health, housing, financial situation, social participation or personal relationships. This could include re-employment. The duration of these programmes varied, with a maximum duration of 1 year.

### Data Collection

A questionnaire was sent to the home address of the participants, followed by two reminders two respectively 4 weeks later. Additional actions were undertaken to include more persons. The questionnaire and covering letter were translated in Turkish and sent in addition to the Dutch questionnaire to persons with a Turkish surname which constitute the largest ethnic minority. If persons needed help with filling in the questionnaire, they could get in touch with an interviewer. Persons who did not reply to the postal questionnaire, were visited by an interviewer at their home address with at least two attempts at different day times during a 2 week period.

### Individual Characteristics

Sociodemographic variables, such as ethnic background, education, age, sex and marital status, were collected by questionnaire. Ethnic background of the respondent was based on the country of birth of the mother. When the mother was born in The Netherlands, the country of birth of the father was used. Different ethnic groups were defined, based on differences in experiences of migration (refugees or labour migrants) and differences in geographical and cultural distance from The Netherlands. Three ethnic minority groups were defined: (1) Turks and Moroccans; (2) Antilleans and Surinamese; and (3) a miscellaneous group with all other countries of origin. Persons were divided into two groups according to the highest level of educational attainment. An intermediate/high educational level was defined as higher vocational training, university, higher secondary schooling or intermediate vocational training; and low educational level was defined as no education, primary school, lower and intermediate secondary schooling or lower vocational training. Marital status was used to distinguish those subjects married or living with a partner from others.

Three psychological factors were collected. Personal mastery was measured at baseline by the Personal Mastery Scale which consisted of six items (e.g., “I have little control over the things that happen to me”, “There is little I can do to change many of the important things in my life”), answered on a five-point Likert scale (strongly agree to strongly disagree) [31]. The sum score of the 6 items was calculated, ranging from 6 to 18; a higher score indicated a higher level of mastery. Self-esteem was measured with the Rosenberg Self-Esteem Scale [32] with 10 items (e.g., ‘‘On the whole, I am satisfied with myself’’, ‘‘All in all, I am inclined to feel that I am a failure’’), answered on a four-point Likert scale (strongly agree to strongly disagree). Average sum scores were calculated, ranging from 10 to 40; a higher score indicated a higher level of self-esteem.

Attitude towards work including motivation was measured with 3 items (e.g., “I am satisfied with my life if I find a job”, “I would like to have a paid job at this moment”) [26]. Sum scores were calculated, ranging from 10 to 40; a higher score indicated a higher attitude towards work.

### Primary Outcome Measures

Participation in paid employment was assessed by questions on the number of hours per week that were spend in paid work. Any paid employment was defined as working for at least 1 h per week. Fulltime paid work defined as working for at least 36 h per week.

Participation in voluntary work was assessed by two questions on the number of times per month a person was actively volunteering (for example at school, in a choir, music association, sports club, hobby club, mosque or church, nursery, etc.). Five answer categories were given: “at least once a week”, “2 or 3 times a month”, “once a month”, “less than once a month”, “never”. Voluntary work was defined as actively volunteering for at least once per month.

### Secondary Outcome Measures

Health was measured with the standardized questionnaire Short-Form Health Survey (SF12) [33]. The 12 items of the SF-12 were used to calculate scores on two dimensions, physical and mental health. Scores could range from 0 to 100, with a higher score indicating a better health.

Anxiety and depressive symptoms were measured with the Kessler Psychological Distress Scale (K10), which consisted of 10 items measuring the level of anxiety and depressive symptoms a person may have experienced in the last 4 weeks. Scores could range from 10 to 50, with a higher score indicating more anxiety and depressive symptoms [34].

### Process Evaluation

During the study 12 semi-structured interviews and two focus group interviews were undertaken with members of the interdisciplinary team (psychologists, employment specialists, social workers) to obtain insight in the fidelity of the implementation of the interdisciplinary re-employment programme. Barriers and facilitators for implementation of the interdisciplinary re-employment programme were discussed. At the end of the intervention programme, semi-structured interviews were undertaken with 10 successful and 10 unsuccessful participants in order to obtain insight into different aspects of the intervention that could be improved in the future, from the point of view of the participants.

### Statistical Analyses

The propensity score is defined as the probability of exposure to the intervention given a number of confounding variables. The propensity score was estimated with logistic regression analysis, modelling the exposure to the interdisciplinary re-employment programme as dependent variable and individual characteristics as independent variables. First, univariate associations between sociodemographic characteristics, health and psychological characteristics with assignment to the intervention were investigated. Variables with a p-value of 0.10 or less were retained in the multivariate model as well as sociodemographic variables by default. In case of a high correlation between two independent variables, the variable with the highest explained variance was included in the multivariate model. For each individual, the likelihood of being exposed to the intervention was estimated with the multivariate regression model and used for covariate adjustment in the GEE analyses [35].

To examine changes in labour force participation and health, repeated-measures regression analyses were used by the generalized estimating equations method. This method takes into account the intra-individual correlation between measurements and is not sensitive to missing measurements. Outcome measures of the repeated regression analyses were fulltime paid employment (≥36 h per week), any paid employment, voluntary work, physical health, mental health and anxiety and depressive symptoms. With the following regression model the change of the outcome measure in time among participants of the interdisciplinary re-employment was compared with the change among participants in regular re-employment programmes.

$${Y_t} = {\beta _0} + {\beta _1}*{\text{grou}}{{\text{p}}_t} + {\beta _2}*{\text{tim}}{{\text{e}}_t} + {\beta _3}*{\text{time}} \times {\text{grou}}{{\text{p}}_t} + {e_t}$$

Here *Y*_*t*_ is the outcome measure of a person at time *t; group* is an indicator variable for the type of programme (intervention programme = 1, regular programme = 0); *time* is a continuous variable indicating time in years from the start of the re-employment programme; *time* × *group* is an interaction term of *time* and *group*. In this model, *β*_0_ estimates the baseline level of the outcome measure at time zero; *β*_1_ estimates the difference of the outcome measure at baseline between the two groups; *β*_2_ estimates the change of the outcome measure per year during the follow-up period (i.e. the baseline trend); and *β*_3_ estimates the change in the trend of the outcome measure in group 1 (intervention group) compared to the trend in group 0 (reference group) The error term *e*_t_ at time *t* represents the random variability not explained by the model.

For the dichotomous outcome measures ((fulltime) paid employment, voluntary work), the betas were transformed in odds ratios that represented the yearly increase or decrease in the likelihood of starting with paid or voluntary work.

Variables with a maximum of 10% missing values were imputed using an iterative Markov chain Monte Carlo (MCMC) method (see additional Table 1) [36]. For the dependent variables it was assumed that not filling in the questions about labour force participation implied that persons were not active on the labour market. Persons who were fulltime employed at baseline (n = 7) were excluded from the analysis.

**Table 1:** Baseline characteristics of persons in the intervention programme and in usual programmes, and contribution to propensity score

	Intervention programme (n = 181)	Usual programmes (n = 253)	Contribution to propensity score OR (95% CI)
Age (mean, sd)	38.1 (7.7)	42.2 (8.4)	0.95 (0.93–0.98)

	N (%)	N (%)	
Sex (women)	108 (59.7)	131 (51.8)	1.18 (0.76–1.84)
Education			
Low	130 (70.8)	199 (78.7)	1
Intermediate/high	51 (28.2)	54 (21.3)	0.77 (0.45–1.30)
Married/living with partner	28 (15.5)	56 (22.1)	
Children	75 (41.4)	115 (45.5)	
Ethnicity			
Native Dutch	46 (25.4)	59 (23.3)	1
Turkish/Moroccan	41 (22.7)	48 (19.0)	1.43 (0.70–2.91)
Surinamese/Antillean	53 (29.3)	76 (30.0)	1.12 (0.62–2.03)
Other	41 (22.7)	70 (27.7)	1.14 (0.58–2.25)
Unemployment duration			
<1 year	39 (21.5)	35 (13.8)	1
1–5 years	72 (39.8)	83 (32.8)	0.60 (0.31–1.14)
>5 years or never worked	70 (38.7)	136 (53.8)	0.41 (0.22–0.76)
Dutch language skills (poor)	47 (26.0)	111 (43.9)	0.42 (0.24–0.73)

	Mean (sd)	Mean (sd)	
Physical health (0–100, higher is better)	54.6 (26.1)	52.1 (27.5)	
Mental health (0–100, higher is better)	44.4 (25.0)	55.5 (25.8)	0.99 (0.98-1.00)
Anxiety and depressive symptoms (10–50, higher is more symptoms)	28.8 (9.1)	25.5 (9.9)	
Mastery (6–18, higher is better)	12.2 (2.7)	13.0 (2.8)	
Self-esteem (10–40, higher is better)	28.1 (4.7)	30.1 (4.9)	0.94 (0.90-1.00)
Attitude towards work (0–10, higher is better)	3.60 (2.3)	3.12 (2.2)	1.07 (0.96–1.18)

Multivariate model: Nagelkerke R^2^ = 0.22

## Results

Figure 1 shows that, at baseline, 869 persons were enrolled in the study after referral to an interdisciplinary re-employment programme (n = 380) or regular re-employment programmes (n = 489). Response to the first questionnaire was 48% (n = 181) among participants of the intervention programme and 52% (n = 253) among participants of the usual programme. Response at follow-up was higher among participants of the usual programmes (n = 107/253; 42%) than among participants of the intervention programme (51/181; 28%). Loss to follow-up was higher among younger (<35 years) participants, but not related to other individual characteristics or (mental) health at baseline.

Table 1 shows that participants of the intervention programme had a younger age, better Dutch language skills, a more positive attitude towards work and were more recently employed than participants of the usual programmes. On the other hand, mental health problems, low mastery, and low self-esteem were more common among participants of the intervention programme compared to the usual programmes.

**Fig. 2:**
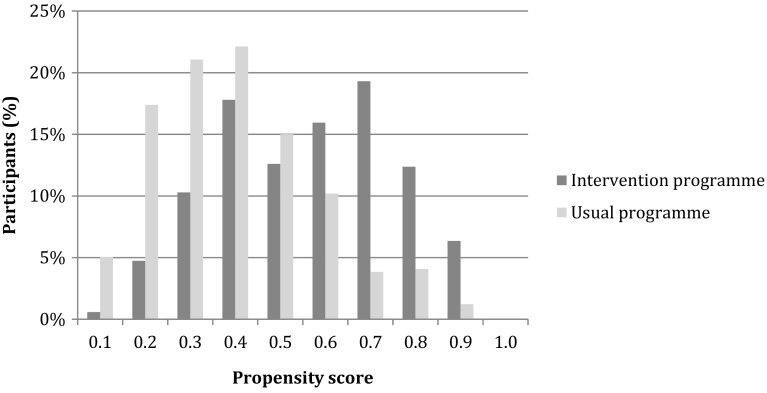
Distribution of the predicted probability of treatment assignment (propensity score) to the intervention programme and usual programmes

The propensity score was estimated for each individual based on sociodemographic factors (sex, age, education, ethnicity) as well as unemployment duration, language skills, mental health, self-esteem and attitude towards work. Figure 2 shows that the propensity score distribution was slightly different for the intervention and usual programmes group, but showed a large overlap.

Table 2 shows a positive 2-yearly change in labour force participation among participants of the intervention programme as well as the usual programme. After 2 years, 10% of the participants of the intervention programme worked fulltime, compared to 4% of the participants of the usual programmes. The percentage of persons working any hours (at least 1 h per week) increased from 9% at baseline to 26% after 2 years among the intervention group, compared to an increase of 8–22% among the usual care group. However, there was no statistically significant difference in increase of labour force participation between the intervention group and the usual care group in the unadjusted model as well as in the propensity score adjusted model.

**Table 2:** Effect of participation in interdisciplinary re-employment programme and regular re-employment programmes on entering fulltime paid employment, any paid employment and voluntary work among unemployed persons

	Start of study % (N)	After 2 years % (N)	Difference in 2-year change intervention versus reference	
			Crude OR(95%CI)	Adjusted OR (95%CI)
Fulltime paid employment (GE36 h/week)				
Interdisciplinary re-employment programme	0%	9.8% (5/51)	1.50 (0.35–6.44)	1.65 (0.38–7.11)
Usual re-employment programme	0%	3.7% (4/107)		
Any paid employment				
Interdisciplinary re-employment programme	9.4% (17/181)	25.5% (13/51)	0.61 (0.26–1.43)	0.69 (0.25–1.87)
Usual re-employment programme	7.9% (20/253)	21.5% (23/107)		
Voluntary work (at least once a month)				
Interdisciplinary re-employment programme	18.8% (34/181)	35.3% (18/51)	1.25 (0.55–2.81)	1.15 (0.45–2.93)
Usual re-employment programme	26.1% (66/253)	42.1% (45/107)		

*Adjusted* propensity score in regression model, *Crude* propensity score *not* in regression model

In addition, a positive 2-yearly change of voluntary work was found among participants of the intervention programme as well as the usual programme. The percentage of persons who did voluntary work (at least once per month) increased from 19% at baseline to 35% after 2 years among participants of the intervention programme, compared to an increase of 26–42% among participants of the usual programmes. There was no statistical difference in increase of participation in voluntary work between the intervention group and the usual care group.

Table 3 shows that the physical and mental health of participants of the intervention as well as the usual programme did not change during the 2-year period. At baseline, participants of the intervention programme had a worse mental health status of (mean 44.4) compared to participants of the usual programme (mean 55.5). During the 2-year period, mental health status remained unchanged among participants of the intervention as well as the usual programme. In addition, there was no significant difference in 2-year change in physical health or anxiety and depressive symptoms among participants of the intervention and usual programme.

**Table 3:** Effect of participation in interdisciplinary re-employment programme and usual re-employment programmes on mental and physical health among unemployed persons

	Start of study Mean (sd)	After 2 years Mean (sd)	Difference in 2-year change Intervention versus Reference	
			Crude Beta (sd)	Adjusted Beta (sd)
Physical health [0–100 (higher is better)]				
Interdisciplinary re-employment programme	54.6 (26.1)	50.2 (27.1)	−4.09 (4.11)	−3.91 (4.11)
Usual re-employment programme	52.1 (27.5)	51.7 (26.2)		
Mental health (0–100, higher is better)				
Interdisciplinary re-employment programme	44.4 (25.0)	44.4 (23.6)	1.51 (4.10)	1.38 (4.15)
Usual re-employment programme	55.5 (25.8)	55.0 (24.4)		
Anxiety and depressive symptoms (10–50 higher is more symptoms)				
Interdisciplinary re-employment programme	28.8 (9.07)	28.0 (9.61)	−0.23 (1.60)	−0.19 (1.57)
Usual re-employment programme	25.5 (9.93)	24.8 (9.65)		

*Adjusted* propensity score in regression model, *Crude* propensity score not in regression model

Table 4 shows an improvement of physical health among persons who entered paid employment (+16%), whereas an improvement of physical health was not found among persons who remained unemployed. Persons who entered paid employment had a better mental health at baseline compared to persons who remained unemployed. Anxiety and depressive symptoms decreased among persons who entered paid employment (−15%), but not among those who continued to be unemployed. Starting with voluntary work was not associated with an improvement of physical health, mental health or anxiety and depressive symptoms during the 2-year follow-up period.

**Table 4:** Change in mental and physical health during a follow-up period of 2 years among persons who were (part-time) employed (n = 37) versus non-employed (n = 397) at the end of the follow-up period

	Start of study Mean (sd)	After 2 years Mean (sd) % change	Difference in 2-year change re-employed versus unemployed Beta (sd)
Physical health (0–100, higher is better)			
Employed (any hours) at the end of the follow-up period of two years	55.6 (31.0)	64.6 (22.0) + 16.2%	14.99 (6.12)*
Continuously unemployed	52.9 (26.6)	47.1 (26.4) −11.0%	
Mental health (0–100, higher is better)			
Employed (any hours) at the end of the follow-up period of 2 years	58.2 (22.2)	60.0 (24.1) +3.1%	2.89 (5.46)
Continuously unemployed	50.1 (26.3)	48.9 (24.2) −2.4%	
Anxiety and depressive symptoms (10–50, higher is more symptoms)			
Employed (any hours) at the end of the follow-up period of 2 years	26.2 (9.4)	22.4 (8.4) −14.5%	−3.67 (2.13)**
Continuously unemployed	27.0 (9.7)	26.9 (9.9) −0.4%	

*Adjusted* propensity score in regression model, *Crude* propensity score not in regression model

*p < 0.05; **p < 0.10

## Discussion

The interdisciplinary re-employment programme did not have a positive influence on re-employment or physical or mental health among unemployed persons with common mental health problems. However, among persons who entered paid employment, physical health improved and anxiety and depressive symptoms decreased, whereas health remained unchanged among persons who continued to be unemployed.

During the 2-year follow-up period of the study, participation in paid employment increased from 10 to 26% among participants of the interdisciplinary programme, which was approximately the same (8–22%) for the regular programmes. After 2 years, 10% of the participants of the intervention programme worked fulltime, compared to 4% of the participants of the usual programmes. However, the statistical analysis could not demonstrate that these observed differences were statistically significant. Therefore, it is concluded that the interdisciplinary re-employment programme has no added value with regard to paid employment compared to the regular programmes. There are three possible reasons why the intervention was not effective: (1) the study could not demonstrate an effect due to methodological limitations, (2) the intervention was not successfully implemented, or (3) the intervention was indeed not effective in this form.

### Methodological Limitations

Because a randomised controlled design was not feasible, the propensity score method was used as an alternative research design to investigate the effectiveness of the interdisciplinary re-employment programme. Because allocation of persons to the intervention and control group was not random, differences between the intervention and control group existed. Based on the observed differences in sociodemographic characteristics and health, the propensity score was calculated. The goal of the propensity score method is to balance two non-equivalent groups on observed covariates to get more accurate estimates of the effects of a treatment. The likelihood of being exposed to an intervention given a set of covariates was estimated with logistic regression analysis and used for covariate adjustment in the analysis of the effect of the interdisciplinary programme. However, unobserved factors that may influence employment or health may have potentially biased the results.

The probability of entering paid employment was low and the observed differences between the intervention and control group were small. Therefore, the absence of a statistically significant difference in labour force participation between the intervention and control group may be due to a lack of power.

### The Intervention was not Successfully Implemented

A process evaluation was done to investigate the fidelity of the re-employment programme. Key elements of the interdisciplinary re-employment programme were (1) high integration of vocational and mental health services, (2) rapid job placement, (3) cognitive counselling, and (4) individual job search support. Integration of vocational and mental health services was enhanced by regular interdisciplinary meetings and mental health specialists working at the employment services. Cognitive counselling was done by the mental health professionals and individual job search support was provided by the employment specialists. However, the process evaluation showed that rapid job placement was not implemented very well, because this was taken care of by another department of the employment services. When participants were ‘ready to start with employment’ they were referred to another department of the employment services. The employment professionals of that department were under high pressure to fill as much vacancies as possible. The unemployed persons with (an history of) mental health problems were substantially less likely to get a job offer due to this organisational structure and the way the professionals of the different departments were directed.

### The Intervention was Indeed not Effective in this Form

The interdisciplinary team predominantly focused on dealing with mental health or social problems before encouraging participants to quickly enter paid employment. This strategy is called the “train then place” method. However, different studies have provided evidence for another strategy, which is called the ‘place then train’ method: the IPS approach for persons with severe mental health problems consists of rapid job placement and job-coaching in the new job [20]. This approach resulted in higher rates of competitive employment, fewer days to the first competitive job and more hours and weeks worked among persons with severe mental disorders [21–23]. However, evaluation studies of the IPS intervention were always done among persons with severe mental problems, whereas participants of the interdisciplinary re-employment programme had common mental disorders, such as anxiety or depression. A Norwegian study among persons with common mental disorders on long-term benefits showed that integration of the IPS principles and work-focused cognitive behavioural therapy (CBT) was an effective strategy to increase work participation [37].

In addition, participants of the IPS intervention were all motivated to seek work, which is an important predictor of entering paid employment [26]. In the current study, attitude towards work was not an inclusion criteria for participation in the interdisciplinary re-employment programme, since all participants were on social benefits with the requirement that they are available for paid employment. This may partly explain the lower proportion of participants of the interdisciplinary re-employment programme entering paid employment compared to the IPS intervention.

Another important element of the IPS intervention is the time-investment of the employment specialist to build up a network of employers who are willing to accept participants with health problems into their company. In the current study, employment specialists of the interdisciplinary team were not allowed to build up a network of potential employers, because this was the responsibility of another department of the employment services. Therefore, the organisational structure may have limited the results with respect to entering paid employment.

The fact that most of the participants did not achieve the primary goal of entering paid employment may have had a negative influence on the mental health of participants. This may explain why the mental health of participants did not improve, despite the fact that they participated in cognitive counselling. However, among those persons who entered paid employment physical health improved and anxiety and depressive symptoms decreased. This was irrespective of their participation in the interdisciplinary or regular programmes.

### Societal Context of the Study

The study was undertaken in the years 2011–2014 during a strong recession in the Netherlands with growing unemployment. Due to the economic situation, there were less opportunities to gain work for unemployed persons in general. It may have been especially difficult for unemployed persons with health problems to be considered for the few available jobs. Thus, unemployed persons with health problems may have considered it not very encouraging to apply for vacancies with a high competition of other applicants with a favourable labour market position.

Due to the social security regulations in the Netherlands, entering part-time paid employment does not always result in a sufficient income that enables the person to be no longer dependent on (additional) benefit. In most situations, entering a part-time job will not result in an increase of personal income as income from paid employment will be deducted from the social benefit. The administrative burden for workers is very high, especially when the job is temporary or has irregular hours. Therefore, the financial incentive to enter paid employment is lacking when employment of at least 32 h is not possible.

### Limitations of the Study

Different strategies were undertaken to improve the response to the questionnaires. The initial response to the postal questionnaire was low (approximately 29%). Therefore, additional action were undertaken to increase the response. An independent researcher provided help with with filling in the questionnaire at the Employment Center or at their home address. This resulted in a total response of approximately 50% of the first questionnaire. Taken into account the characteristics of the study population this is a fair response.

Another limitation of the study was the large variation in content and activities of the interdisciplinary re-employment programme. A review showed that challenges of complex interventions include the standardisation of interventions, the impact of people involved and the organisational context of implementation [38]. Therefore, it is difficult to characterize specific components that contributed to positive outcomes.

## In Conclusion

The interdisciplinary re-employment programme did not have a positive influence on re-employment or physical or mental health among unemployed persons with health problems. However, among persons who entered paid employment, physical health improved and anxiety and depressive symptoms decreased, whereas among persons who continued to be unemployed health remained unchanged. Policies to improve population health should take into account that promoting paid employment may be an effective intervention to improve health.

Inclusion of unemployed persons with common mental health problems in the workforce through re-employment programs is an important strategy to improve their health. It is recommended to include rapid job placement and coaching on the job (first place then train method) as part of an interdisciplinary re-employment programme. In addition, it is of paramount importance to take into account the needs of employers and invest in building a network of potential employers, as part of the re-employment process of unemployed persons with mental health problems.

## Electronic supplementary material

Below is the link to the electronic supplementary material.

Supplementary material 1 (DOCX 15 KB)
