# Supplementary material for: Influence of an Interdisciplinary Re-employment Programme Among Unemployed Persons with Mental Health Problems on Health, Social Participation and Paid Employment
Source: J Occup Rehabil. 2017 Apr 10;28(1):147–57. doi: 10.1007/s10926-017-9704-3 (PMC5820402; doi:10.1007/s10926-017-9704-3)
Supplement: Supplementary file 1 — Supplementary material 1 (DOCX 15 KB) [file 10926_2017_9704_MOESM1_ESM.docx]

Additional table 1 Imputation of missing values

|  | Original data | | | Missing% | Imputed data | | |
| --- | --- | --- | --- | --- | --- | --- | --- |
|  | ExiT | Ref | Total | Total | ExiT | Ref | Total |
| Age (mean, sd) | 186 | 255 | 441 | **0** | 186 | 255 | **441** |
| Sex (women) | 186 | 255 | 441 | **0** | 186 | 255 | **441** |
| Education  - low  - intermediate / high | 180 | 236 | 416 | **5.7** | 186 | 255 | **441** |
| Married/living with partner | 176 | 241 | 417 | **5.4** | 186 | 255 | **441** |
| Children | 176 | 241 | 417 | **5.4** | 186 | 255 | **441** |
| Ethnicity  - Native Dutch  - Turkish/Moroccan  - Surinamese/Antillean  - Other | 179 | 244 | 423 | **4.1** | 186 | 255 | **441** |
| Unemployment duration  <1 year  1-5 years  >5 years or never worked | 179 | 245 | 424 | **3.9** | 186 | 255 | **441** |
| Dutch language skills (poor) | 175 | 242 | 417 | **5.4** | 186 | 255 | **441** |
| Physical health (0-100)  (higher is better) | 184 | 252 | 436 | **1.1** | 186 | 255 | **441** |
| Mental health (0-100)  (higher is better) | 184 | 248 | 432 | **2.0** | 186 | 255 | **441** |
| Anxiety and depressive symptoms (10-50) High is more symptoms | 173 | 229 | 402 | **8.8** | 186 | 255 | **441** |
| Mastery (6-18) (higher is better) | 170 | 233 | 403 | **8.6** | 186 | 255 | **441** |
| Self-esteem( 10-40) (higher is better) | 166 | 227 | 393 | **10.9** | 186 | 255 | **441** |
| Attitude towards work (0-10) (higher is better) | 171 | 227 | 398 | **9.8** | 186 | 255 | **441** |
| Fulltime paid employment (≥36 hours/week) | 164 | 218 | 382 | **13.4** | 186 | 255 | **441** |
| Parttime paid employment (12-35 hours/week) | 164 | 218 | 382 | **13.4** | 186 | 255 | **441** |
| Paid employment (any hours) (>0 hours per week) | 164 | 218 | 382 | **13.4** | 186 | 255 | **441** |
| Voluntary work (at least once a month) | 176 | 242 | 418 | **5.2** | 186 | 255 | **441** |
